# Immature teratoma presenting as a soft-tissue mass with no evidence of other sites of involvement: a case report

**DOI:** 10.1186/s13000-016-0527-x

**Published:** 2016-08-15

**Authors:** Radamés Ádamo Zuquello, Giordano Tagliari, Rodrigo Bagatini, Ricardo Hohmann Camiña, Ruggero Caron, Nadia Aparecida Lorencette, Antuani Rafael Baptistella, Gabriel Manfro

**Affiliations:** 1 Universidade do Oeste de Santa Catarina, Joaçaba, Brazil; 2 Hospital Universitário Santa Terezinha, Joaçaba, Brazil; 3 Oncology research group of Hospital Universitário Santa Terezinha/Universidade do Oeste de Santa Catarina, Joaçaba, Brazil; 4 Department of Clinical Oncology, Hospital Universitário Santa Terezinha, Joaçaba, Brazil; 5 Programa de Pós-Graduação em Biociências e Saúde/Universidade do Oeste de Santa Catarina, Joaçaba, Brazil; 6 Department of Oncological Surgery, Hospital Universitário Santa Terezinha, Joaçaba, Brazil; 7 Travessa Domingos Bonato, 37 – CEP: 89600-000 Joaçaba, Santa Catarina/SC Brazil

**Keywords:** Germ cell tumor, Teratoma, Soft tissue

## Abstract

**Background:**

Germ cell tumors are tumors composed of tissues derived from more than one of the three germinal layers. They are more common in the testes and ovaries, but can present in many different regions in the midline, including the sacral region, retroperitoneum, mediastinum, and brain. Testicular germ cell tumors generally metastasize to the retroperitoneum, lungs, and brain; metastases to soft tissue are very rare.

**Case presentation:**

Here we describe a case of a single soft-tissue mass in the thigh of a 27-year-old man, with histology showing areas of mature teratoma tissues derived from the ectodermal and mesodermal lineages, and areas of immature teratoma tissue composed of small undifferentiated cells, with primitive neuroectodermal differentiation foci forming neuroepithelial elements – thus classified as immature teratoma. The patient had no other clinical or radiological evidence of involvement, besides the lymph nodes.

**Conclusion:**

The case presented suggests a rare and unexpected primary immature teratoma of the thigh.

## Background

Teratoma is a subtype of germ cell tumors (GCT) derived from more than one of the three germinal layers. Teratomas can be classified as mature tumors (cystic or solid), which contain well-differentiated tissues, or as immature tumors, which contain poorly differentiated tissues consisting primarily of embryonic-appearing neuroglial or neuroepithelial components [1, 2]. The most commonly involved sites of teratomas are the sacrococcygeal region (57 %) and the gonads (29 %); they occur much more frequently in the ovaries, but can also arise in the testes. In adults, the gonads are by far the most common site of teratomas. Other possible locations are the mediastinum (7 %), retroperitoneum (4 %), cervical region (3 %), and intracranial (3 %) [3]. Uncommon locations are the stomach, heart, pleura, pharynx, thyroid, base of the skull, maxilla, liver, prostate, vagina, and subcutaneous tissues [2, 4].

GCTs represent 95 % of testicular tumors developing after puberty, although pure teratomas of the testis are rare (3–5 %). In men, teratomas of the testis developing during and after puberty are always considered to be malignant because of the potential for metastasis, mainly to the retroperitoneal lymph nodes [3]. According to Ghazarian et al. [5], almost 90,000 cases of testicular GCTs in men were registered in the US between 1998 and 2011.

The prognosis of teratomas depends on many factors. Mature teratomas are generally benign. Immature teratomas in young children also tend to behave as benign tumors. In patients older than 15 years, immature teratomas can manifest as highly devastating malignancies [1].

Metastases of testicular teratomas to the subcutaneous tissues are very rare. Only two cases of primary testicular teratomas that metastasized to soft tissue (the left thigh and gluteal and iliac muscles, respectively) have been reported in the literature [6, 7]. In addition, the literature contains only four descriptions of GCTs arising primarily from structures away from the midline [8–11]. Here we present the first reported case of an immature teratoma manifesting as a subcutaneous mass in the thigh, with no evidence of other sites of involvement, except the lymph nodes.

## Case presentation

**Fig. 1:**
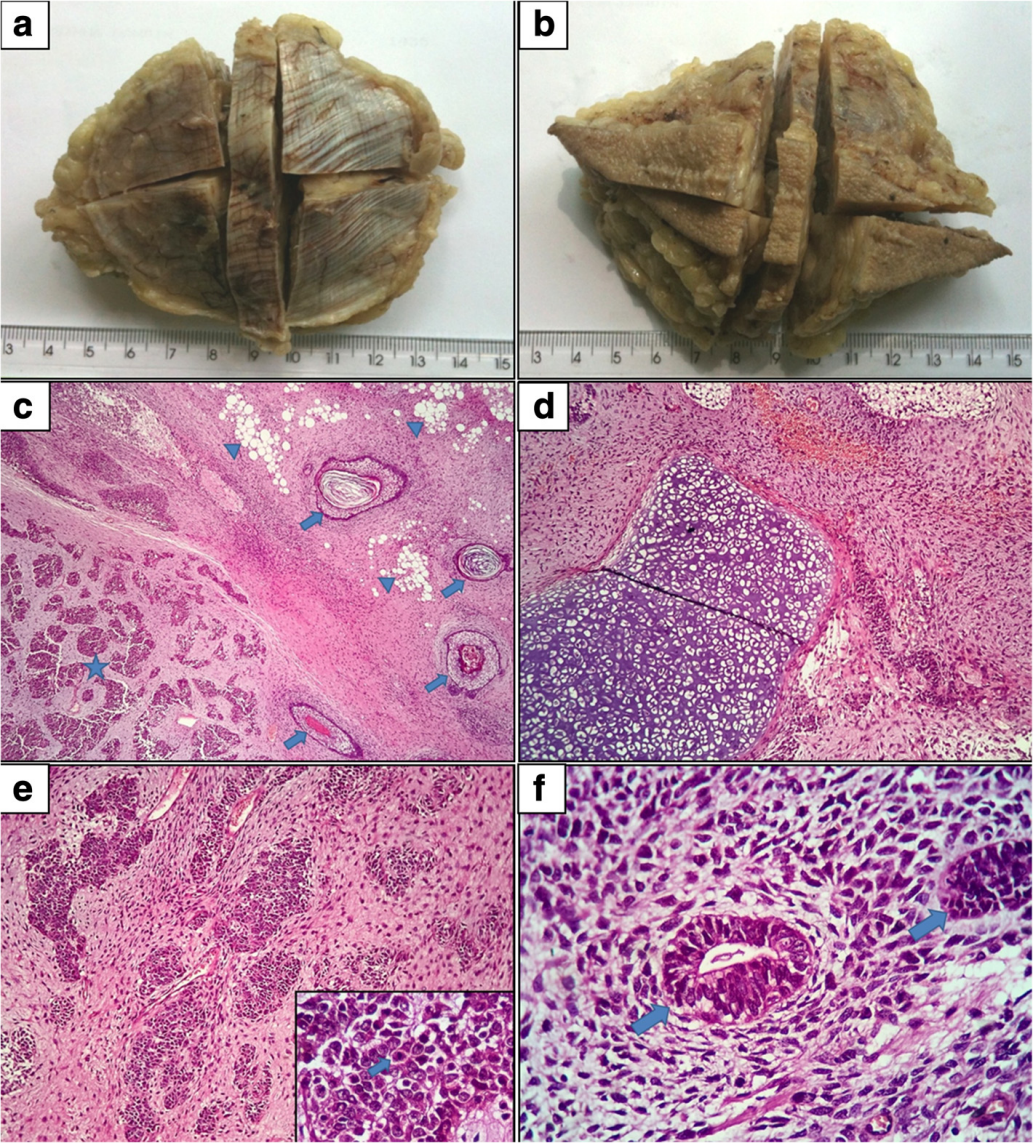
Gross specimen of the tumor, after the first surgery, with muscle fascia (**a**) and epidermis side (**b**). **c** Histological section showing immature teratoma (left), represented by blocks of undifferentiated neoplasm (asterisk). On the right, areas of mature teratoma composed of well differentiated tissue with corneal cysts (arrows) and islands of adipose tissue (arrowhead). (Hematoxylin & Eosin, 40× magnification); **d** Island of cartilaginous tissue of mesodermal lineage (mature teratoma), surrounded by undifferentiated cells (immature teretoma), (Hematoxylin & Eosin, 100× magnification); **e** areas of immature teratoma composed of blocks of undifferentiated neoplasm. In the lower picture, small undifferentiated cells with mitotic figures (arrows), (Hematoxylin & Eosin, 100× magnification, and lower frame 400× magnification); **f** Immature teratoma composed of small undifferentiated cells with foci of primitive neuroectodermic differentiation forming neuroepithelial elements (arrows), (Hematoxylin & Eosin, 400× magnification)

A 27-year-old man presented to his city’s health service (in the Midwest of the state of Santa Catarina, Brazil) in February 2010 complaining of a nodule in the middle third of the lateral aspect of his right thigh. The mass was removed surgically and sent for pathological examination, which revealed a 105 g piece, measuring 10.5 × 8.5 × 4 cm, covered by white skin. In the central portion of dermis/hypodermis, there was a brownish, lobulated, soft, and friable nodule, measuring 6.5 × 5 × 3.5 cm, partially encapsulated. The deep margin was covered by whitish and smooth muscle fascia (Fig. 1a and 1b). Histological sections stained with hematoxylin-eosin showed mature and immature teratoma, with presence of areas of mature teratoma with tissues derived from ectodermal and mesodermal lineages (Fig. 1c and 1d), and areas of immature teratoma composed of small undifferentiated cells (Fig. 1e) with foci of primitive neuroectodermic differentiation forming neuroepithelial elements (Fig. 1f). The prevalence of immature teratoma was estimated to be 70 %. Margins were compromised, and a sample was sent for immunochemical analysis.

**Fig. 2:**
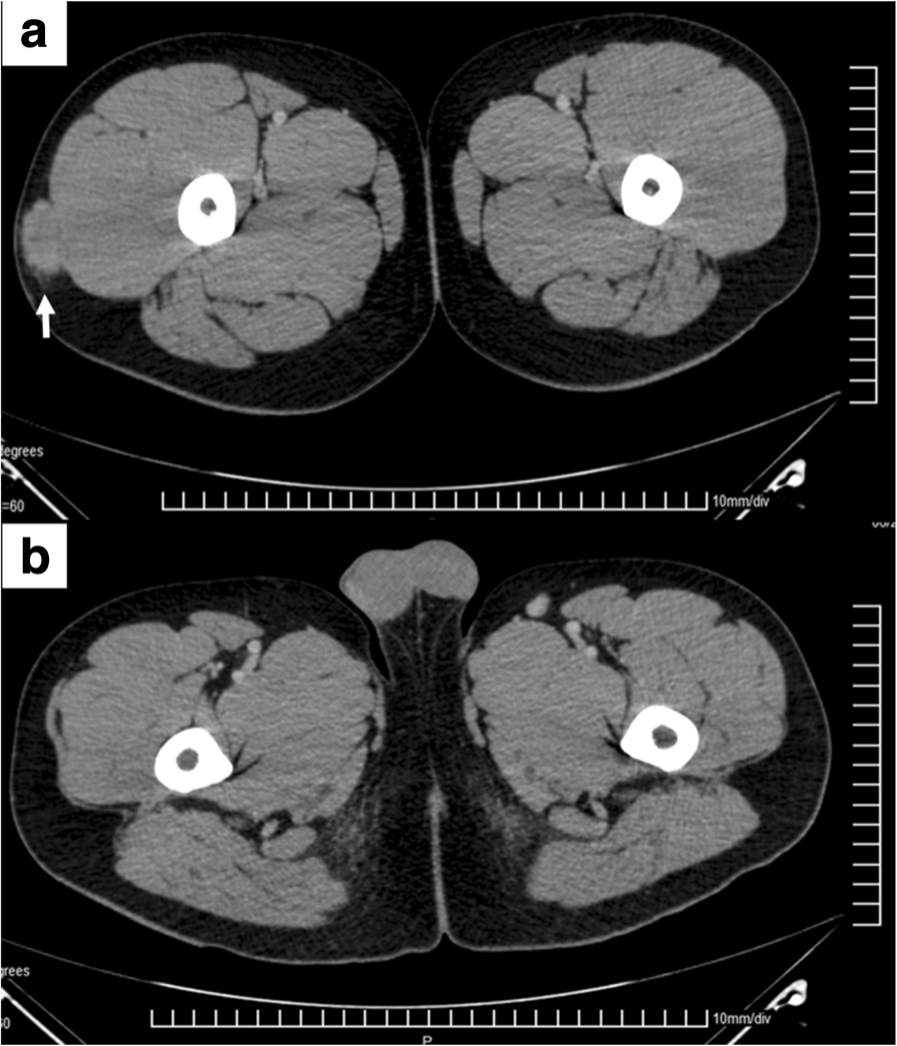
**a** Axial CT image of the inferior extremities shows a muscle-density mass in the lateral aspect of the right thigh, measuring 38 × 20 mm (arrow). **b** Axial CT image of the perineum at the testes level shows the lack of disease in these structures

While waiting for the immunochemical results, the patient was referred to the Oncology Service of Hospital Universitário Santa Terezinha (HUST). He presented at this service in May, 2010 with a hard nodule (diameter ~5 cm) in his right thigh, in the same location from which the previous mass was removed. Computed tomography (CT) of the thigh revealed a mass in the lateral aspect of the thigh (Fig. 2a). Findings of chest CT and chest radiography, conducted as part of the workup to assess possible sarcoma, were negative. The testes were also normal, as determined by CT (Fig. 2b).

Immunochemical results, available in June 2010, demonstrated positivity for cytokeratin (AE1 and AE3 clones), CD99 and MIC2 (Ewing’s sarcoma and 12E7 markers), PS100 (anti-human S-100), Wilm’s tumor 1 (WT1, 6 F-H2 clone), desmin (D33 clone), and vimentin (V9 clone), consistent with an immature teratoma. The patient underwent a second surgery to remove the mass. Analysis of the second resection specimen revealed a yellowish, irregular node, partially encapsulated and greasy to the touch, weighing 24 g and measuring 4.8 × 3.8 × 1.8 cm. Surgical margins were free and there was no perineural or angiolymphatic invasion. The patient was scheduled for follow up.

In November 2010, the patient presented to the Oncology Service of HUST with a right inguinal node. Ultrasound of the testis and CT of the brain were normal. CT of the pelvis and abdomen revealed enlarged lymph nodes, measuring up to 2.7 cm, in the right inguinal region and retroperitoneum. No other abnormalities were seen. The patient’s alpha-fetoprotein (AFP) level was 375.7 ng/ml (normal value < 15 ng/ml, slightly varying among laboratories) and his β–human chorionic gonadotropin (β-hCG) level was 0.1 mIU/ml (normal value < 2,67 mIU/ml, slightly varying among laboratories). At this point, the patient was started on bleomycin, etoposide, and cisplatin, and was followed regularly. The right inguinal node regressed.

In May 2011, the patient presented to the Oncology Service of HUST with right inguinal pain. Physical examination revealed no right inguinal lymph node or testicular abnormality. The patient returned in June 2011 with right inguinal enlargement, up to 2.5 cm. Serum levels of AFP and β-hCG were 5.6 ng/ml and 0.1 mIU/ml, respectively.

The right inguinal lymph node was biopsied in August 2011 and sent for immunochemical analysis. This analysis revealed positivity for FLI-1 (Friend leukemia integration 1 transcription factor), (MRQ-1) and synaptophysin (anti-synaptophysin, SY38 clone) and negativity for thyroid transcription factor 1 (SPT24 clone), WT-1 (6 F-H2 clone), PS100 (anti-human S-100), CD99 and MIC2 (12E7 clone), cytokeratin (AE1 and AE3 clones), chromogranin A (DAK-A3 clone), and desmin (D33 clone). Laboratory findings received in September 2011 showed that the patient’s AFP level was 35.6 ng/ml and his β-hCG level was 2.3 mIU/ml. The patient was started on rescue chemotherapy with paclitaxel, ifosfamide, and cisplatin in February 2012. Thereafter, concentrations of the tumor markers AFP and β-hCG were 5.6 ng/ml and 0.6 mIU/ml, respectively.

In March 2012, the patient presented with pain in his right thigh. His AFP level was 61.4 ng/ml and his β-hCG level was 4.8 mIU/ml. CT of the thigh revealed recurrence of the mass. Right inguinal node enlargement was also noticed.

In May 2012, the patient returned to the Oncology Service of HUST with a palpable node in his left breast, in addition to the inguinal and thigh nodules. In June 2012, his AFP level was 1,101.8 ng/ml and his β-hCG level was 1.5 mIU/ml. The patient was started on palliative chemotherapy with the VIP (vinblastine, ifosfamide, and cisplatin) protocol. By the end of June 2012, after the first cycle of VIP, the patient developed neutropenic fever, septic shock, acute kidney injury and finally died in consequence of these complications.

## Discussion

The case presented in this article is very rare, and it is the first reported case of an immature teratoma in the subcutaneous tissue of the thigh with no evidence of a primary tumor site. As this case is unusual, many questions arise. The first question is whether the tumor arising in the subcutaneous tissue was a teratoma, rather than a sarcoma. Our answer is that the immunochemical findings were clear and concise. The positivity for all immunochemical markers and the elevation of characteristic serological tumor markers allow us to have no doubt about the histologic type of the tumor. Common immunochemical markers for sarcoma are vimentin, keratin, desmin, leucocyte common antigen, and S100 [12], but not CD99 and MIC2, which characterize primitive neuroectodermal components, a hallmark of immature teratomas [13, 14]. The second question is how an immature teratoma can first present in the subcutaneous tissue of the thigh, with no evidence of a primary tumor. We propose two theories to explain this fact. First, an undetected primary tumor may have been unable to grow in its original site, leading to clinical evidence of only metastatic disease. Second, the thigh may have been the primary site of the tumor.

We would like to emphasize the absence of findings in our patient’s brain, chest, pelvic, and abdominal CT, with positivity only for the retroperitoneal and inguinal lymph nodes. In addition, ultrasound of the testes was normal, and physical examination of the four extremities and neck were also normal.

We found in the literature two reported cases of immature teratomas with soft-tissue metastases, interestingly, to the thigh and the gluteal region [6, 7]. Both cases involved clear primary gonadal tumors, in contrast to our case. We also found descriptions of four cases of GCTs arising outside of the midline, without evidence of a primary tumor; the authors considered these GCTs to be primary tumors [8–11]. One of these reports describes a malignant mixed GCT in the soft tissue of the right arm of a 37-year-old man, with no other sites of involvement; in this case, immunochesmistry was not performed, and serum markers for GCT were within normal limits [8]. Another report describes the case of a malignant teratoma in the left proximal humerus of a 14-year-old girl. Also in this case, no other sites of disease were found [9]. In this case, immunochemical findings were consistent with a GCT. An extragonadal malignant teratoma of the foot [10] and an intraosseous teratoma of the ilium [11], both without evidence of other sites of involvement that could suggest a primary tumor, have also been described.

Teratomas originate from germ cells, which first appear in the endoderm of the yolk sac and then migrate to the genital ridges, through the wall of the midgut, during the fifth week of gestation. The abnormal migration of germ cells in the intrauterine period can lead to GCTs in extragonadal locations. Our patient had normal, topic testes. Although ectopic testes can be found in the medial thigh [15], we found no evidence in the literature that they can be located in the lateral aspect. We thus assume that the tumor in this case was not gonadal in origin.

The lower limb begins to grow in the fourth week of embryonic development, arising from the sacral region opposite the fifth lumbar and first sacral somites. At the 6–9-mm stage, in approximately the fourth gestational week, the limb bud lengthens and the base extends toward the sacral myotomes [16–18]. The sacrococcygeal region, from which the lower limb arises, is one of the most common locations for immature teratoma development, especially in infants. We speculate that germ cells in the sacrococcygeal region can become trapped and follow the lower limb during its development in this case.

During the course of the disease, our patient developed inguinal and retroperitoneal lymph node enlargement. This drainage route is consistent with dissemination from the thigh, first to the superficial and deep inguinal nodes and then to the external iliac and aortic nodes. Lymphatic drainage of the testes occurs first to the interaortocaval and left para-aortic lymph nodes, just below the renal vessels (as classically seen in metastatic GCT of the testes). Thus, we hypothesize that the lymph node metastases in this case likely originated from the thigh [19–21].

If we assume that the mass in the thigh was secondary to a primary occult tumor (e.g., testicular or retroperitoneal), it likely developed through hematogenous dissemination (as retrograde lymphatic dissemination is very unlikely), though it is uncommon for a GCT. Bilici et al. [7] reported a case of a stage IA immature teratoma of the testis that was treated surgically. The tumor relapsed years after treatment, with, among others, a mass in the thigh that was proven histologically to be an immature teratoma. In that case, however, the patient also had multiple lung, liver, mediastinal, and brain metastases, rather than the single metastasis that characterizes our case [7].

Soft-tissue metastases of a solid tumor are generally uncommon; it usually occurs in the setting of advanced, relapsed malignancy [7, 21]. On the other hand, Damron and Heiner stated that metastatic soft-tissue masses present most commonly before or concomitant with the primary malignant sites [22]. Contrary to that statement, in this case, we have a single soft tissue mass, which hardly could represent a metastatic mass, since no evidence of other site of involvement was found.

## Conclusions

The case presented here is challenging and unique. None of the hypothesis that we have developed to explain it – either a soft-tissue metastasis as the initial presentation of an immature teratoma arising in an unknown primary site or a primary immature teratoma arising in the thigh from germ cells sequestered abnormally in a location never previously described – matches evidence in the literature. The first hypothesis – a single metastasis to soft tissue with no evidence of disease in any organ except the lymph nodes – could be considered more probable, given the premise that GCTs do not arise outside of the midline. We found only two reported cases in which teratomas spread to soft tissue, but definite primary sites were identified in both cases. In the present case, the testes may have been the primary site of the tumor; after a single metastasis to the subcutaneous tissue of the thigh, the original lesion may have undergone spontaneous necrosis and was no longer clinically evident. The second hypothesis, that the soft-tissue mass was primary, is supported by four other described cases of GCTs outside the midline with no evidence of any other disease site. Attention should be paid to similar cases in the future, to achieve a better understanding of the behavior of GCTs, especially teratomas.

## References

[1] Helman LJ, Malkin D. Cancers of the Childhood. In: DeVita, Hellman, and Rosenberg, editors. Cancer: Principles and Practice of Oncology, Philadelphia: Lippincott Williams & Wilkins; 2011

[2] Isaacs H (2013). Germ cell tumors. Tumors of the fetus and infant: an atlas.

[3] Hamilton CA, Ellison MC. Cystic Teratoma. Medscape Medical Reference. 2015. http://emedicine.medscape.com/article/281850-overview. Accessed 21 Aug 2015

[4] Sachveda K et al. Extragonadal Germ Cell Tumors. Medscape Medical Reference. 2015. http://emedicine.medscape.com/article/278174-overview. Accessed 21 Aug 2015

[5] Ghazarian AA et al. Incidence of testicular germ cell tumors among US men by census region. Cancer. 2015. doi: 10.1002/cncr.29643 10.1002/cncr.29643 PMC4666746 26280359

[6] Husband JE, Bellamy EA. Unusual thoracoabdominal sites of metastasis in testicular tumors. American Journal of Roentenology. 1985. http://www.ajronline.org/doi/abs/10.2214/ajr.145.6.1165 10.2214/ajr.145.6.1165 2998169

[7] Bilici A et al. Case report: Soft tissue metastasis from immature teratoma of the testis: second case report and review of the literature. Clin Orthop Relat Res. 2010. doi: 10.1007/s11999-009-1173-3 10.1007/s11999-009-1173-3 PMC2919860 19937408

[8] Benali (2012). Extragonadal mixed germ cell tumor of the right arm: description of the first case in the literature. World J Surg Oncol.

[9] Koh JS, Park JH, Kang CH (2009). A primary extragonadal teratoma of the proximal humerus. J Korean Med Sci.

[10] Chinoy (1992). Extragonadal malignant teratoma of the foot. Indian J Cancer.

[11] Vazquez (2000). Intraosseous teratoma of the iliac bone. Pediatr Radiol.

[12] Singer S, Maki RG, O’Sullivan B. Soft tissue sarcoma. In: DeVita, Hellman, and Rosenberg, editors. Cancer: Principles and Practice of Oncology, Philadelphia: Lippincott Williams & Wilkins; 2011

[13] Husain N, Verma N (2011). Curent concepts in pathology of soft tissue sarcoma. Indian J Surg Oncol.

[14] Morovic A, Damjanov I (2008). Neuroectodermal ovarian tumors: a brief overview. Histol Histopathol.

[15] Pugach JL, Steinhardt GF (2002). Evaluation and management of ectopic penile testis. Urology.

[16] O’Rahilly R, Müller F, O’Rahilly R, Müller F (2001). Lower limb. Human embryology and teratology.

[17] Moore KL, Dalley AF, Moore KL, Dalley AF (2005). Lower limb. Clinically oriented anatomy.

[18] Mooney EK, Loh C. Lower limb morphology, Gross morphologic overview of lower limb development. Medscape Medical Reference. 2013. http://emedicine.medscape.com/article/1291712-overview. Accessed 21 Aug 2015

[19] Bosl GJ et al. Cancer of the testis. In: DeVita, Hellman, and Rosenberg, editors. Cancer: Principles and Practice of Oncology, Philadelphia: Lippincott Williams & Wilkins; 2011

[20] Moore KL, Dalley AF, Moore KL, Dalley AF (2005). Abdomen. Clinically Oriented Anatomy.

[21] Plaza JA (2008). Metastases to soft tissue: a review of 118 cases over a 30-year period. Cancer.

[22] Damron TA, Heiner J (2000). Distant soft tissue metastases: a series of 30 new patients and 91 cases from the literature. Ann Surg Oncol.

